# Multiparametric Material Functionality of Microtissue‐Based In Vitro Models as Alternatives to Animal Testing

**DOI:** 10.1002/advs.202105319

**Published:** 2022-01-18

**Authors:** Elena Stengelin, Julian Thiele, Sebastian Seiffert

**Affiliations:** ^1^ Department of Chemistry Johannes Gutenberg‐University Mainz D‐55128 Mainz Germany; ^2^ Leibniz‐Institut für Polymerforschung Dresden e.V. Hohe Straße 6 D‐01069 Dresden Germany

**Keywords:** 3R principle, hydrogels, in vitro models, microtissue engineering

## Abstract

With the definition of the 3R principle by Russel and Burch in 1959, the search for an adequate substitute for animal testing has become one of the most important tasks and challenges of this time, not only from an ethical, but also from a scientific, economic, and legal point of view. Microtissue‐based in vitro model systems offer a valuable approach to address this issue by accounting for the complexity of natural tissues in a simplified manner. To increase the functionality of these model systems and thus make their use as a substitute for animal testing more likely in the future, the fundamentals need to be continuously improved. Corresponding requirements exist in the development of multifunctional, hydrogel‐based materials, whose properties are considered in this review under the aspects of processability, adaptivity, biocompatibility, and stability/degradability.

## Graphical abstract

Overview of microtissue‐based in vitro model systems as a possible substitute for animal testing according to the 3R principle from Russel and Burch is presented herein. Special attention is given to fabrication techniques, to natural and synthetic materials, as well as to multifunctional material properties, such as processability, adaptivity, biocompatibility, and stability/degradability.

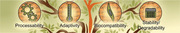

## 1 Introduction

Animal testing is a common approach in industry and academia to classify the risks and effects of pharmaceutics, pesticides, biocides, and food additives on the environment and humans.^[^ ^1^ ^]^ However, their use simultaneously raises questions as well as pros and cons of moral and ethical nature that have been the subject of controversy over decades.^[^ ^2^, ^3^ ^]^

Provoices argue based on the physiological similarity of animals and humans. As a result, conclusions are drawn regarding the effect of tested substances in animal experiments on the human organism, which has already made it possible to classify a large number of substances in terms of danger and benefit to the society.^[^ ^4^ ^]^ By contrast, voices against animal experiments argue from an ethical as well as scientific and economic point of view. Ethically, the question generally arises as to why human welfare should have a higher priority than animal welfare. This topic is not the subject of this review, and we refer to more detailed accounts in this regard, such as those by Petetta et al. and Ferdowsian et al.^[^ ^2^, ^5^ ^]^ From a scientific point of view, animal experiments are often considered a black box whose results are based on functions and mechanisms that are challenging to understand.^[^ ^3^, ^6^ ^]^ This lack of knowledge may result in erroneous transferability to the human organism, especially because influencing factors such as gender, age, occupation, lifestyle, and disease are not taken into account, which is one of the reasons why only few substances successfully pass the clinical phase.^[^ ^3^, ^7^ ^]^ Moreover, the interlaboratory reproducibility of animal studies is low.^[^ ^7^ ^]^ From an economic standpoint, animal experiments are resource‐intensive (time‐consuming and costly) and require skilled labor.^[^ ^8^ ^]^ For example, drug approval takes 10–15 years.^[^ ^7^, ^9^ ^]^

Based on that controversy, Russel and Burch took up the issue of animal testing in 1959 and wrote *The Principles of Humane Experimental Technique* to improve the situation of animals in animal experiments as well as the quality and reproducibility of scientific and medical research.^[^ ^10^ ^]^ Since then, especially in the last two to three decades, there have been significant developments on political and legal levels that have taken up and further optimized Russel and Burch's ideas into internationally accepted ethical frameworks.^[^ ^11^ ^]^ In Europe, for example, the *EU Directive 86/609/EEC* was founded in 1986 to which each member state has to comply.^[^ ^12^ ^]^ In 2010 it was revised, resulting in the currently valid guideline *EU Directive 2010/63/EU*, which is a basis for regulations in the individual EU states.^[^ ^2^, ^13^ ^]^ In the United States, animal testing is controlled by the Animal Welfare Act since 1966.^[^ ^2^ ^]^ At the heart of all these regulations, Russel and Burch's so named 3R principle forms the benchmark for scientific quality and ethical considerations. In that abbreviation, 3R stands for Refinement, Reduction, and Replacement of animal experiments.^[^ ^14^ ^]^ Based on that premise, when animal experiments are necessary, inhumane procedures should be avoided and animal welfare should be improved (Refinement), while the number of experimental animals should be reduced to a minimum (Reduction).^[^ ^11^ ^]^ In addition, intensive research should be carried out on alternatives to replace animal experiments involving living vertebrates (Replacement).^[^ ^8^ ^]^

**Figure 1:**
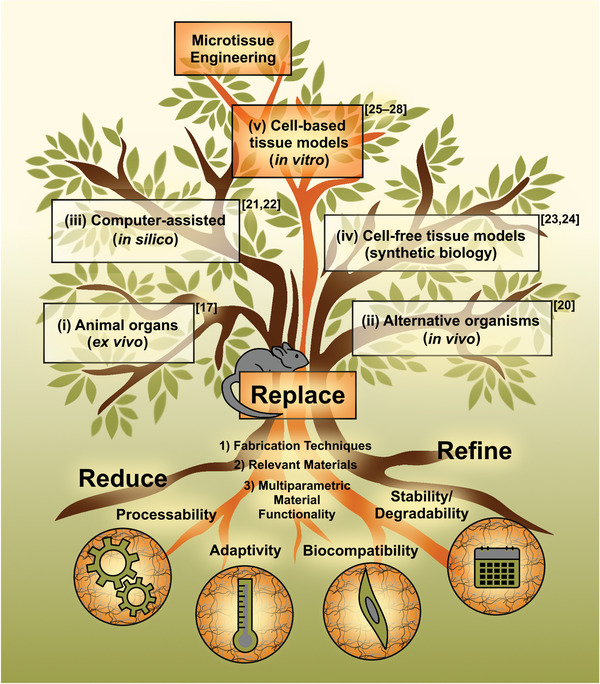
Schematic of a tree with a key pathway highlighted in orange starting at the roots and ending in the crown to illustrate the theme and story line of this review. Based on the 3R principles (reduce, replace, and refine), the review's focus is on the replacement of animal experiments through cell‐based microtissue models. Accordingly, fabrication techniques 1), relevant hydrogel‐based materials 2), and multiparametric material functionality 3) are addressed as the main foundation for the cell‐based microtissue alternatives (“crown”) with particular focus on material processability, adaptivity, biocompatibility, and stability/degradability (“roots”). In addition, other potential alternatives to animal testing are highlighted through the branches of the tree, such as animal organs, alternative organisms, computer‐assisted technologies, and cell‐free tissue models. Since these will not be discussed in detail in the remainder of this review, reference is made to selected literature collections.

Overall, the 3R principle covers many disciplines and areas. Refining and reducing animal testing primarily focuses on medical and regulatory areas, whereas replacing animal testing focuses on a broader range of research topics, as illustrated in **Figure** **1** using the branches of a tree to illustrate the spreading of this concept.^[^ ^1^, ^8^, ^15^ ^]^ In branch (i), ex vivo procedures are situated, which focus on animal organs (e.g., skin) outside the living organism, while in (ii), alternative organisms such as the invertebrates (e.g., Drosophila) and the fish embryo test are listed.^[^ ^16^, ^17^, ^18^, ^19^, ^20^ ^]^ Other alternatives are based on iii) computer‐assisted in silico methods for drug discovery, iv) cell‐free tissue models (e.g., imitation of complex organisms as cells), and v) cell‐based tissue or in vitro models (2D and 3D cell cultivation).^[^ ^21^, ^22^, ^23^, ^24^, ^25^, ^26^, ^27^, ^28^ ^]^ In this article, we focus on cell‐based tissue alternatives, as highlighted in Figure 1, whereas we refer to selected reviews for the others, as quoted just before.

An excellent path toward cell‐based tissue models is microtissue engineering, as it addresses the complexity of natural tissues on a microscopic level, but in a simplified form.^[^ ^29^, ^30^ ^]^ Appropriately engineered models aim to replicate only specific areas and functions of the human organism, enabling new, intelligent and specific preclinical testing methods applicable to any specific human situation and also allowing for precisely monitoring system processes.^[^ ^3^, ^31^ ^]^ The foundation of such microtissue engineered models is based on suitable cells and well‐designed scaffold structures, with the chemical and structural composition of the shaping materials in particular guiding the model systems. The more functional the materials are, the more complex and intelligent alternatives can be achieved. Hence, this review presents research on the multiparametric material properties of cell‐based microtissue models as a substitute for animal testing. It includes 1) an overview of fabrication techniques, 2) a selection of relevant hydrogel‐based materials, and 3) their multiparametric material functionality. The latter focuses on the main hydrogel requirements that are *processability, adaptivity, biocompatibility*, and *stability/degradability* (Figure 1).

In many of the publications covered in this article, the original context is actually more on aspects like tissue engineering and drug delivery, whereas they in fact often not explicitly refer to the replacement of animal testing; nevertheless, despite their originally different designation, these studies may also address aspects of 3R as well. This review aims at reflecting these studies in view of this topic.

## 2 Fabrication Techniques

The increasing complexity in structure and function of artificial microtissue is closely related to the advancements in design and engineering of materials processing strategies and methods. The following section will discuss a selection of these processes based on *bioprinting*, *spheroids*, *microfluidics*, and *organ‐on‐a‐chip*, that have advanced the design of functional cocultures, microtissues, and organoids (**Table** **1**).

**Table 1:** Comparison of bioprinting and microfluidic technology

	Properties^a)^	Current research challenges	Application examples
Bioprinting	Basis: hydrogels, suitable cells/spheroids, and bioprinters Shear‐thinning, fast gelling, form and mechanically stable, biocompatible hydrogels/bioinks desirable^[^ ^33^ ^]^ µm–mm–cm scaling; low throughput	Multimaterial printing; adaptive and responsive culture systems; vascularization of tissue; bioprintable material availability; on‐demand production^[^ ^48^ ^]^	Printing of microstructures with embedded cells/spheroids for, e.g., the imitation of in vivo tissues^[^ ^40^ ^]^
Microfluidics	Basis: hydrogels, suitable cells/spheroids, and microfluidic devices Simple flow behavior of hydrogels desirable; form and mechanically stable, biocompatible hydrogels desirable; oxygen and nutrient exchange by flow cells µm scaling; size tunability and uniformity; direct characterization; low throughput	High throughput fabrication; automation, integration, and intelligent synthesis of biomaterials^[^ ^49^ ^]^	Template‐mediated spheroid synthesis for, e.g., tissue formation^[^ ^43^ ^]^ Organ‐on‐the‐chip applications for, e.g., toxicity and efficacy testing^[^ ^47^ ^]^

^a)^ The table is intended to provide an exemplary overview but does not claim to be complete.

*Bioprinting* is an additive‐manufacturing process that make use of cells in media as well as in to‐build‐up tissue structures in a bottom‐up approach. The material basis of bioprinting is commonly known as bioinks.^[^ ^32^ ^]^ Bioinks are usually based on bioprintable hydrogels with shear‐thinning properties, fast gelation times, and shape retention properties, that are also capable of entrapping cells.^[^ ^33^ ^]^ The broader application of bioprinting for microtissue design requires two major foundations: suitable cell material and bioprinters at an advanced level of engineering, e.g., to not harm living cells during bioink processing by mechanical forces induced by the printing process itself.^[^ ^34^ ^]^ Only then we can sufficiently address the complex parameter space of minimal feature size (resolution), vascularization, perfusion, automation, cost, diffusion of molecules, growth factors and nutrients as well as the supply of mechanical and biochemical stimuli. Although bioprinting has been shown to be able to create microstructures with embedded cells, it requires more than that. The key properties of an in vivo environment—(multi‐)cellular assemblies with dense cell–cell or cell‐extracellular matrix (ECM) interactions—are additionally required to approximate the structure and biochemistry of the native environment of cells and tissues. Only these have the potential to provide a new set of tools for understanding diseases and the effectiveness of patient‐specific therapies, while being based on human cells, such models may be eventually more predictive than animal models, thus reducing or even replacing the need for animal testing.

A key element in designing such multiparametric, multicellular platforms could involve the use of *spheroids*.^[^ ^35^ ^]^ These densely packed microtissue units can be formed template‐free or engineered by the support of microparticles, e.g., polymer microgels, which have also emerged as individual engineered cell scaffolds themselves (cf. below).^[^ ^36^ ^]^ While traditional tissue scaffolding follows a top‐down approach, e.g., based on implants or transplants,^[^ ^37^ ^]^ the concept of bottom‐up construction of microtissue by spheroids holds great promises for the design of multiphasic cell matrices with tissue‐specific structures across scales.^[^ ^38^ ^]^ Exemplarily, Torisawa et al. utilized a continuous‐flow microfluidic device equipped with a semiporous membrane to regulate culture media flow toward distinct geometric compartments, which then filled with cocultures. In there, weeks‐long culturing yielded self‐aggregated, individual spheroids with microtrap‐controlled size and shape.^[^ ^39^ ^]^ And Mekhileri et al. precisely placed spherical microstructures into 3D‐plotted scaffolds (bottom‐up approach) using computer‐assisted layer‐by‐layer bioprinting and promoted the growth of large and complex tissues with improved architectural control. Compared to the top‐down approach, this strategy has advantages in cell–cell interaction and natural cell arrangement in the tissue due to the preprogrammed composition.^[^ ^40^ ^]^ While the design of single cell line based spheroids is rather straight‐forward, the orchestration of multiple cell lines requires platforms that provide compartmentalization *and* spatial control over matrix conditions for optimal coculturing. A solution to that could be the usage of core–shell (polymer) microstructures. For instance, the groups of Park, Shin and coworkers established the sequential seeding of mesenchymal stem cells (MSCs) and endothelial cells on individual hydrogel patches made from a thermoresponsive hydrogel. Upon thermal actuation, these hydrogel structures transitioned into core–shell spheroids that, after an additional fusion step, lead to the formation of vascularized microtissue.^[^ ^41^ ^]^

Spheroids can be utilized as building blocks in tissue design due to the large parameter space of potential template (e.g., material basis, size, shape, functionalization, and elasticity) and tissue properties (e.g., cell line and cell density). However, for fabricating physiological microtissue constructs based on human cells, some of the above‐discussed methods lack material uniformity (e.g., of spheroids and their templates). They also do not provide the necessary fabrication rates and ability to validate and characterize these in a high‐throughput fashion.^[^ ^42^ ^]^ On this account, Matsunaga et al. established the *microfluidic* high‐throughput production of uniform collagen microparticles via axisymmetric flow‐focusing devices as templates for cell overgrowth and spheroid formation.^[^ ^43^ ^]^ Using NIH 3T3 cells, HepG2 cells, human umbilical endothelial cells (HUVECs), primary neurons, primary rat hepatocytes, and MIN6m9 cells, the versatility of this template‐mediated spheroid formation was highlighted. Microgels with seeded cells adhered to each other and eventually fused via cell–cell interactions coated on the collagen gel beads. The cells also grew into the collagen gel beads, which eventually lead to gel decomposition and macroscopic tissue formation. In fact, microfluidics generally is the most‐established method for producing microemulsions that act as templates for uniform hydrogel‐based microstructures.^[^ ^44^, ^45^ ^]^ The power of microfluidic‐based material design lies in its ability to tune the size of biocompatible soft microtissue from a few to hundreds of micrometers, whose physicochemical and mechanical properties can be approximated to that of the native ECM. Yet, for closely mimicking mechanobiological cues of the ECM as well as regulating the ECM's effect on cell differentiation and migration, artificial microtissue design also requires the processing of ECM molecule mixtures or ECM‐derived materials. In continuous‐ and segmented‐flow (droplet‐based) microfluidics, this task is often challenging, as mixtures of polysaccharides and proteins (containing collagen, fibronectin, laminin, hyaluronic acid, among others), that are essential ingredients of the natural ECM, may exhibit complex flow behavior. Exemplarily, shear‐thinning of these multicomponent mixtures may exacerbate the throughput of material in microfluidic devices as, in the case of droplet formation, only low flow rates will provide stable droplet formation.^[^ ^46^ ^]^

Microfluidic devices do not only serve as enabling technology for the design of tissue building blocks and artificial niches, as discussed above, but their exact control overflow pattern formation in microchannels with tailored‐made architecture and integrated functional units (e.g., valves, micropumps, membranes, vents, and hydrodynamic traps) is most suitable to control cell attachment (e.g., in microstructured niches), cell agglomeration with spatiotemporal control, nutrient and gas flow. Based on well‐established manufacturing methods of microfluidic devices including combined photo‐ and soft lithography, additive manufacturing based on fused deposition modeling and projection microstereolithography, and glass microcapillaries, such platforms have evolved as experimental platform in cell biology, e.g., for mimicking tissue organization and its physiological environment. Researchers have pushed this development toward so‐called *organs‐on‐a‐chip*, which contain human‐derived cells preserved with biophysical and chemical cues to mimic the structure and function of single human organs and even interconnected organs embedded in microfluidic systems. Functioning as simplified organ models, they enable a wide range of in vitro toxicity and efficacy testing. Such microfluidic devices can be used in place of animals or animal‐derived tissue models to replicate human physiology in disease research, testing of drug safety or the effect of chemicals, cosmetics, and consumer products on human tissue in the admission process. For example, Purtscher et al. have integrated a dual cell culture bioassay into a common lab‐on‐a‐chip platform for evaluating the safety of pharmaceutical products.^[^ ^47^ ^]^ Key examples of these microfluidic‐based experimental platforms for microtissue design have made the transition into commercialization. For instance, AlveoliX's platform emulates the complexity of the human lung, named lung‐on‐a‐chip, or tissue barriers, and MIMETAS’ microfluidic cell culture plates provide perfused tubular tissues in a parallelized fashion without the presence of artificial membranes. Beyond microfluidic platforms, Swedish Fluicell has developed a 3D bioprinting system with micrometer precision for medical research models in cell dish and microtiter plates that potentially complement and decrease animal testing, e.g., in intermediate stages of drug development.

## 3 Relevant Materials

**Figure 2:**
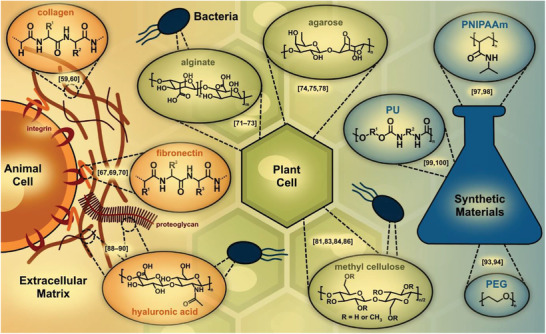
Schematic overview of some relevant natural materials obtained from animal, plant, and bacterial sources, as well as some representative synthetic materials.

In addition to the material design, the materials themselves are also an important building block (or even the most important one) for the design of in vitro model systems. Materials commonly used in this context are hydrogels, e.g., physically or chemically crosslinked 3D polymers that swell in aqueous media.^[^ ^50^, ^51^ ^]^ In the following, a selection of natural and synthetic materials is presented and explained in terms of their structural composition, accessibility and origin, as schematically shown in **Figure** **2**. There are many more bio‐based hydrogel types and we refer to much more detailed reviews by Thiele et al., Van Vlierberghe et al., Caliari et al., and Rice et al.^[^ ^52^, ^53^, ^54^, ^55^ ^]^

### 3.1 Natural Materials

The main representatives of natural materials are based on proteins and polysaccharides derived from animal, plant, and bacterial sources. Proteins and polysaccharides are commonly part of the ECM where they are scaffold and promoter of cell–cell/cell‐matrix interactions and of cellular activities.^[^ ^56^, ^57^ ^]^

An essential natural protein of the ECM is *collagen type I*, which is composed of *n*‐alternating amino acid sequences [–Gly–X–Y–]*_n_* with glycine (Gly) and any amino acid X and Y linked by amide bonds [—CO—NH—].^[^ ^58^, ^59^, ^60^ ^]^ In natural ECM tissue, three such polypeptide chains arrange in a triple helix and connect to each other by hydrogen bonding. These triple helices stack together and connect covalently by lateral interactions to form fibrils, which in turn aggregate to larger fibers.^[^ ^52^, ^61^ ^]^ Through solubilization of collagen fibers (e.g., collagen type I from rat‐tail tendons) at enzymatic and salt/acid conditions, collagen fibrils are extracted and become applicable for in vitro experiments.^[^ ^54^, ^62^, ^63^ ^]^ By hydrolysis or denaturation of collagen type I, *gelatin*, an alternative protein based on single‐strand molecules, can be obtained.^[^ ^53^, ^55^, ^64^, ^65^, ^66^ ^]^ Another type of ECM proteins are multiadhesive glycoproteins (a combination of proteins and polysaccharides), that contain several binding domains for interacting with the ECM matrices, cell surface receptors, and other glycoproteins.^[^ ^67^ ^]^ A prominent representative is *fibronectin*, which primarily connects ECM matrices to cell adhesion receptors (integrins).^[^ ^68^ ^]^ It consists of two similar, intramolecularly linked polypeptide subunits (230–270 kDa), whose assembly of type I, type II, and type III repeating units is responsible for collagen/gelatin and integrin specificity.^[^ ^67^, ^69^, ^70^ ^]^

Beside proteins, polysaccharides are also part of the ECM. They are based on a high number of glycosidically linked monosaccharides and obtained from plant, bacterial, and animal sources. A relevant representative, frequently used in food and pharmaceutical industry is *alginate*, a linear polysaccharide, which can be obtained from brown algae (Phaeophyceae) through the treatment with an alkaline solution, or by bacterial synthesis.^[^ ^54^, ^71^ ^]^ Alginate is composed of several (1,4)‐linked *β*‐D‐mannuronic acid (M block) and *α*‐L‐guluronic acid (G block) units, based on [–OH] and [–COOH] functionality, whose molecular weight depends on the source and the fabrication process, typically varying between 10–1000 kDa.^[^ ^53^, ^72^, ^73^ ^]^ An alternative polysaccharide is *agarose*, which is obtained from agar, an posed of (1,3)‐linked *β*‐D‐galactose and (1,4)‐linked *α*‐L‐3,6‐anhydrogalactose, primarily equipped with [–OH] functionality and molecular weight of almost 12 kDa.^[^ ^74^, ^75^, ^76^, ^77^, ^78^ ^]^

The most naturally occurring biomaterial is cellulose, which can be obtained from bacterial and plant sources by chemical treatment.^[^ ^79^, ^80^ ^]^ On a molecular level cellulose is composed of linear (1,4)‐linked *β*‐D‐glucopyranosyl molecules with [–OH] functionality.^[^ ^81^ ^]^ These polysaccharides stack parallel during biosynthesis to fibrils, which in turn aggregate to larger microfibrils with crystalline and amorphous regions, promoted by intermolecular physical interactions.^[^ ^79^, ^82^ ^]^ In general, cellulose is insoluble in water, but this can be overcome by etherification of hydroxyl groups.^[^ ^79^ ^]^ A corresponding known and Food and Drug Administration (FDA)‐approved derivative is *methyl cellulose*, a partially methylated [–O–CH_3_] derivative of cellulose at its hydroxy functionalities with a degree of substitute between 1.7 and 2.0.^[^ ^81^, ^83^, ^84^, ^85^ ^]^ The nonpolar methoxy groups disturb hydrogen bonding between other hydroxy molecules, allowing water molecules to enter the polysaccharide structure, resulting in increased water solubility.^[^ ^86^ ^]^

Considering the ECM environment, there exists a further special linear type of polysaccharides (glycosaminoglycans), which are based on the repeating disaccharides uronic acid and D‐galactosamine or D‐glucosamine.^[^ ^87^ ^]^ *Hyaluronic acid* is its most prominent representative, commercially available and widely used in industry since 1970.^[^ ^88^, ^89^, ^90^ ^]^ It is composed of *β*‐1,4‐D‐glucuronic acid and *β*‐1,3‐*N*‐acetyl‐D‐glucosamine and contains three functional groups [–COOH], [–OH], and [–NHCOCH_3_].^[^ ^88^ ^]^ Hyaluronic acid can be extracted from animal sources or synthesized through bacterial fermentation (*Escherichia coli*), which provides high reproducibility of molecular weight (100–8000 kDa).^[^ ^54^, ^88^, ^91^ ^]^ If the glycosaminoglycans are bound to proteins, proteoglycans are obtained, which serve as further stabilizing components of the ECM.^[^ ^92^ ^]^

### 3.2 Synthetic Materials

Despite practicable properties such as cell adhesion and biodegradability, animal‐derived materials often have poor mechanical properties and batch‐to‐batch variations.^[^ ^66^, ^92^ ^]^ Since their use is also controversial in terms of replacing animal testing, the research is focusing on the development of synthetic materials that cover the entire spectrum of tunable chemical, physical, mechanical, and biological properties. Some synthetic materials are already widely used in microtissue engineering, and three prominent examples are briefly described below.

One of them is *poly*(*ethylene glycol*) (*PEG*), a FDA‐approved polymer, based on repeating ethylene glycol units [–(CH_2_CH_2_O)*_n_*].^[^ ^93^ ^]^ PEG polymers can be generated by ring‐opening polymerization, starting from ethylene oxide. It is commercially available in various molecular weights. In addition, a variety of PEG‐polymer structures are known, such as linear, multiarmed and hyperbranched. Thereby, the designation of PEG usually changes to poly(ethylene) oxide above a molecular weight of 30 kDa.^[^ ^94^ ^]^ Thus, with appropriate end‐group functionalization, PEG‐based hydrogels with diverse mechanical properties are accessible, making them ideal candidates for in vitro applications.^[^ ^92^ ^]^

A second prominent synthetic polymer is *poly*(*N‐isopropylacrylamide*) (*PNIPAAm*), first reported in 1968.^[^ ^95^ ^]^ It is composed of *N*‐isopropylacrylamide monomer units, which are based on hydrophilic amide [–CO–NH–] and hydrophobic isopropyl [–CH(CH_3_)_2_] moieties.^[^ ^96^ ^]^ It has thermoresponsive properties and therefore finds versatile use in tissue engineering, biosensing and drug delivery applications.^[^ ^97^ ^]^ Additionally, PNIPAAm polymers and 3D networks are accessible via numerous synthesis routes as described in detail by Doberenz et al.^[^ ^98^ ^]^

A third type of relevant synthetic materials is based on *polyurethanes* (*PU*), which are widely used in industry since 1937, developed by Otto Bayer and co‐workers. Their characteristic repeating unit is the urethane group [–NH–CO–O–], which classically results from the polyaddition reaction between polyols and polyisocyanates with at least two or more hydroxyl [–OH] and isocyanate groups [—N═C═O].^[^ ^99^, ^100^ ^]^ There are two PU families, thermoplastics and thermosets, which differ in structural design. Thermoplastic PUs have a linear structure and are based on diols, diisocyanates, and chain extenders (low molecular weight diol components), while thermoset PUs form 3D networks based on polyols and polyisocyanates.^[^ ^99^ ^]^ Depending on the application, versatile materials with a wide range of mechanical properties can be obtained.

## 4 Multiparametric Material Functionality

Microtissue‐derived in vitro models are promising candidates to replace animal testing, as they aim to replicate small compartments of the host organ for preclinical studies, rather than focusing on the entire, highly complex organism. The models are based on simply constructed platforms whose components can mimic the extensive tissue properties such as 3D anatomy, functionality, and physiology (e.g., oxygen and nutrient exchange, vascularization, etc.). Because of these properties, they provide an excellent basis for modeling specific organ diseases, modular tissue engineering, and drug delivery studies—all applications where animal testing can potentially be avoided in the future. In addition, due to their microscopic size and synthetic background, the model systems enable the performance of many parallel experiments, a high degree of reproducibility, and the simultaneous performance of analyses during experiments. These features are mainly applied in high‐throughput drug screening and in the analysis of natural processes. Overall, the potential applications of microtissue‐based in vitro model systems are very diverse and require different shapes, as well as a different material base depending on the objectives. Accordingly, different fabrication techniques for providing microtissue platforms, as well as hydrogel‐based materials have been described in the previous sections. To gain a deeper insight into the requirements of hydrogels for microtissue applications, they are discussed in the following sections in terms of their processability, adaptivity, biocompatibility and stability/degradability (**Table** **2**). Finally, an outlook on advanced materials is given and their potential use in in vitro models as a substitute for animal testing is discussed.

**Table 2:** Characteristic chemical groups of selected natural and synthetic materials relevant to the field of microtissue engineering and their multiparametric functionality in terms of processability, adaptivity, biocompatibility, and stability/degradability

	Structure	Processability	Adaptivity	Biocompatibility	Stability/degradability
	Chemical functionality^a)^	Self‐assembly	Modulus *G*′ or *E*	Cell adhesion	Natural degradation
Collagen	[–CO–NH–] [–H, –R]	Physical: pH = 7 (37 °C)	*G*′ = 0.5–429.7 Pa (0.5–5.0 mg mL^−1^)^[^ ^124^ ^]^ *E* = 5–25 kPa (1.0–2.5 mg mL^−1^)^[^ ^212^ ^]^	(+) “direct”	Enzymatic (collagenase)
Gelatin	[–CO–NH–] [–H, –R]	Physical: *T*_UCST_ = 25–30 °C	*E* = 179 Pa (10%)^[^ ^156^ ^]^ *E* ≈ 10 Pa (5%)^[^ ^213^ ^]^	(+) “direct”	Enzymatic (collagenase)
Fibronectin	[–CO–NH–] [–R]	No relevance	No relevance	(+) “direct”	Enzymatic
Alginate	[–OH] [–COOH]	Physical: Ca^2+^, Ba^2+^, Fe^3+^	*E* = 3.6 kPa (1%)^[^ ^157^ ^]^	(−)	Enzymatic (lyase); Solubilization
Agarose	[–OH]	Physical: *T*_UCST_ = 20–70 °C	*E* = 3.6 kPa (1%)^[^ ^157^ ^]^ *E* = 20.2 ± 0.5 kPa (5 wt%)^[^ ^214^ ^]^	(−)	Enzymatic (agarase); solubilization
Methyl cellulose	[–OH] [–O–CH_3_]	Physical: *T*_LCST_ = 40–50 °C	no relevance	(−)	Enzymatic (cellulase); solubilization
Hyaluronic acid	[–COOH] [–OH] [–NHCOCH_3_]	Physical: entangle	*E* = 1.5 kPa (1%)^[^ ^157^ ^]^	(−)	Enzymatic (hyaluronidase); solubilization
PEG	[–(CH_2_CH_2_O)*_n_*]	No relevance	*G*′ = 20.9–30.3 kPa^[^ ^138^ ^]^ *G*′ = 0.1–7.2 kPa 2–5% (w/v)^[^ ^137^ ^]^	(−)	(−)
PNIPAAm	[–CO–NH–] [–CH(CH_3_)_2_]	Physical: *T*_LCST_ = 32 °C	*E* = 86–330 kPa (25.3–37.2 °C)^[^ ^153^ ^]^	T > T_VPTT_ (+) T < T_VPTT_ (−) “indirect”	(−)
PU	[–NH–CO–O–]	(Physical)	*E* ≈ 0.01–0.40 MPa^[^ ^154^ ^]^ *E* = 7.5–2525 Pa^[^ ^155^ ^]^	(−)/(+) “indirect”	Hydrolysis; enzymatic

^a)^ The table is intended to provide an exemplary overview but does not claim to be complete.

### 4.1 Material Requirements

#### 4.1.1 Processability: How to Get Gelation under Control?

The multiparametric functionality of hydrogels is based, among other factors, on their processability, which in the broadest sense refers to their application in manufacturing techniques for shaping. Depending on their intended application, spheroids, microstructures, and modular tissues need to be formed by different manufacturing methods, the use of which depends on the processability of the hydrogels. Extrusion‐based bioprinting, for example, requires shear‐thinning, fast gelling, and form stable materials, as illustrated by the example of Kang et al., who synthesized spheroidal and tubular microstructures by one‐step bioprinting to create human tissue analogs.^[^ ^101^ ^]^ In contrast, the preparation of cell‐loaded microgels by microfluidics requires precursor materials that covalently bond with each other in a time‐delayed manner, e.g., by external stimuli such as UV irradiation, to ensure homogeneous droplet formation.^[^ ^102^ ^]^ Overall, these two technologies show how different and diverse the requirements for the processability of hydrogels are. Accordingly, a selection of natural and synthetic materials will be discussed below regarding their gelling properties, which have a significant influence on their processability. In addition, possible control elements will be addressed.

**Figure 3:**
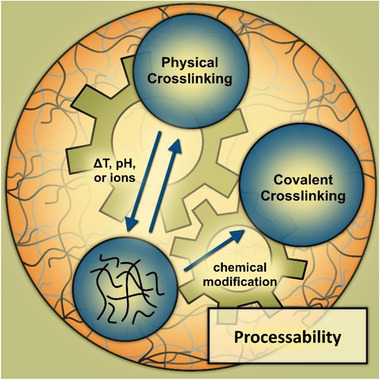
Processability of materials with emphasis on gelling properties and potential control.

In general, most materials gel and form either physically or covalently crosslinked hydrogels as shown schematically in **Figure** **3**. However, for the physically crosslinked hydrogels, external parameters such as temperature, pH and ion concentration often affect the stability of the corresponding gels, which then degrade. This often limits applications in in vitro models due to limited control of gelation time, gel stability, and handling. To overcome this limitation and extend processing options, chemical modification of individual materials is a versatile approach to enable covalent and controllable gelation.

A reversible gelation process of polymers in water is based on temperature and pH changes and depends on the solubility of these polymers in water, as conceptually described by Seiffert et al.^[^ ^103^ ^]^ The temperature at which this solubility changes is either referred to as the lower critical solution temperature (LCST) or to the upper critical solution temperature (UCST). In the former case polymers intermolecularly interact with water molecules at low temperatures through hydrogen bonding and dipole–dipole interactions. However, as the temperature increases, these bonds break, and the polymer chains precipitate in a coil‐to‐globule transition.^[^ ^103^ ^]^ An appropriate example is *PNIPAAm*, whose polar amide groups interact by hydrogen bonding with water molecules below the LCST (32 °C) and precipitate above this temperature.^[^ ^95^ ^]^ Due to that property and the LCST being close to the physiological temperature range, PNIPAAm polymers are widely used as drug delivery materials and beyond.^[^ ^104^ ^]^ To overcome the temperature‐dependent stability of PNIPAAm polymers, they can be copolymerized with functional monomers (e.g., crosslinkers) that make stable hydrogels accessible through chemical or physical interactions.^[^ ^105^ ^]^ Exemplary, Kim et al. have developed multistimuli‐responsive microfibers and microtubes as potential cell scaffolds for tissue engineering applications. These scaffolds are based on NIPAAm molecules, the crosslinker *N*,*N′*‐methylenebis(acrylamide), and the comonomers allylamine or sodium acrylate, which connect covalently by UV irradiation in a microfluidic approach.^[^ ^106^ ^]^

Another thermoreversibly gelling polymer is *methyl cellulose* (LCST = 40–50 °C), whose hydrophobic methyl groups [–CH_3_] self‐assemble into fibrils with increasing temperature and percolate into networks.^[^ ^84^, ^107^ ^]^ Due to that reason, methyl cellulose is usually fluidic at physiological conditions and hence useful as a thickening agent for spheroid synthesis.^[^ ^108^ ^]^ Accordingly, Wang et al. have developed core–shell microgels for organoid synthesis using microfluidics. The microgels consisted of a core based on liquid methyl cellulose and encapsulated liver cells (HepG2) or a coculture of liver and endothelial cells (HepG2/HUVECs) with a stabilizing shell of gelatin methacrylate (GelMA).^[^ ^109^ ^]^ Due to the good printability of methyl cellulose at room temperature but the lack of crosslinking ability, it is also frequently used with polymers such as GelMA and alginate in blend‐based bioinks with high‐dimensional stability and fidelity.^[^ ^110^, ^111^ ^]^

In contrast, in other polymer systems, such as agarose and gelatin, intra‐ and intermolecular polymer–polymer interactions are promoted for enthalpic reasons, which counteract polymer dissolution at low temperatures. By increasing the temperature, these interactions can be overcome, and the polymers dissolve in water at the UCST.^[^ ^103^, ^112^, ^113^ ^]^ For example, *agarose* assembles into double helices and aggregates into ordered structures below its UCST of 20–70 °C and liquefies at higher temperatures.^[^ ^53^, ^114^, ^115^ ^]^ Hence, stable agarose gels for microtissue applications can be obtained at physiological conditions, as described by Struzyna et al. In the corresponding study, agarose‐based microcolumns were filled with proteins and dopaminergic neuronal aggregates to mimic the nigrostriatal pathway for the treatment of Parkinson's disease.^[^ ^116^ ^]^ *Gelatin*, in turn, assembles into triple helices from random coil chains at about 25–30 °C and associates into 3D networks.^[^ ^50^, ^53^, ^114^ ^]^ Since its UCST is below the physiological temperature, they do not form stable gels at 37 °C. Because of this property, it is often used as a sacrificial substrate. For example, Hwang et al. have prepared cell‐laden porous alginate hydrogels by incorporating gelatin microspheres (150–300 µm) that are stable at low temperatures and liquify under physiological conditions.^[^ ^117^ ^]^ To provide temperature‐stable gelatin hydrogels, they are usually functionalized with crosslinking moieties, e.g., with methacrylate groups (known as GelMA).^[^ ^118^ ^]^ A relevant example was provided by Zoratto et al. who developed thermostable microporous scaffolds based on photo‐crosslinked GelMA microgels, to better mimic the ECM and facilitate nutrient and oxygen transport of in vitro models.^[^ ^119^ ^]^ Alternatively, crosslinkers such as glutaraldehyde and genipin can be incorporated and stabilize gelatin networks.^[^ ^120^, ^121^ ^]^

Protonation or deprotonation of pH‐sensitive polymers can further impair the intermolecular polymer–water interaction, which also affects solubility.^[^ ^103^ ^]^ Accordingly, acidic *collagen type I* solutions must be neutralized, causing the triple‐helical fibrils to self‐assemble into fibrillar matrices at about 37 °C within a slow gelation time of half an hour.^[^ ^54^, ^114^ ^]^ A related approach was used by Ugolini et al., who separately confined cell‐laden type I collagen and fibrin gels in PDMS‐based templates to mimic complex biological compartments.^[^ ^122^ ^]^ Further, the gelation of collagen type I is reversible below the denaturation temperature of approximately 37 °C, but gels still remain statically stable for extended periods of time.^[^ ^123^ ^]^ However, to make gelation temperature‐independent and to accelerate the gelation time, chemical modifications are possible that correspond to those described for gelatin.

In turn, gelation of *alginate* occurs with multivalent cations such as Ca^2+^, Ba^2+^, and Fe^3+^, with mainly G‐block elements associating with these to form tight junctions in an egg carton pattern due to negatively charged [–COOH] groups.^[^ ^63^, ^72^ ^]^ This association occurs instantaneously by cations diffusing from the external environment into the alginate precursor solution.^[^ ^72^ ^]^ Agarwal et al. used this approach by preparing cell‐laden core–shell microgels (collagen–alginate) as a biomimetic platform for high‐throughput drug screening. For this purpose, microgels were synthesized by microfluidics, where Ca^2+^ ions (from CaCl_2_) diffused from the oil phase into the alginate precursor droplets and initiate gelation.^[^ ^124^ ^]^ Because alginate gelation is fast, the rate can be slowed down by cations released into the system in a controlled manner, which is called the internal gelation method.^[^ ^72^, ^125^ ^]^ Accordingly, Weitz and coworkers encapsulated MSCs in alginate microgels as possible in vitro model systems for drug testing. For this purpose, they used a precursor solution of alginate and calcium‐ethylenediaminetetraacetic acid (EDTA), which released Ca^2+^ ions at acidic conditions (pH 5) and initiated controlled alginate gelation.^[^ ^126^ ^]^ Alternatively, also CaCO_3_ could be used.^[^ ^127^ ^]^

There are also polymers that do not naturally gel due to external stimuli. Therefore, they must be functionalized to be interesting for microtissue applications. A relevant example is *hyaluronic acid*, which at very low concentrations assumes a rigid helical configuration due to intermolecular hydrogen bonding. These chains entangle randomly, resulting in jelly‐like solutions.^[^ ^128^ ^]^ Chemical modification at the side groups [–COOH], [–OH], and [–NHCOCH_3_] is the basis to form stable covalent‐crosslinked hydrogels. A corresponding example is based on microstranded bioinks by Kessel et al., where gel formation was achieved by photo‐crosslinking of hyaluronan methacrylate. The resulting hydrogels were extruded through a lattice with an aperture size of 40 and 100 µm, resulting in microstrands with shear‐thinning properties that mimic key ECM features.^[^ ^129^ ^]^ For more chemical modifications of hyaluronic acid, we refer to Collins et al. and Khunmanee et al.^[^ ^130^, ^131^ ^]^

Another polymer that also requires prefunctionalization for crosslinking is *PEG*, as it has an LCST of ≈100 °C in water and therefore does not self‐assemble naturally below that temperature.^[^ ^132^ ^]^ A well‐known modification of PEG precursor polymers used in microtissue engineering is the functionalization with acrylates, which enables fast, UV‐induced crosslinking.^[^ ^133^, ^134^ ^]^ Slightly slower and controlled gelation times, in turn, can be achieved by strain‐promoted alkyne azide cycloaddition (SPAAC) and thiol‐ene Michael addition reaction between thiols and vinylsulfones, acrylates/methacrylates, or norbornene‐functionalized PEG polymers.^[^ ^135^, ^136^, ^137^, ^138^, ^139^, ^140^, ^141^ ^]^

*PUs* (usually thermoplastic PUs) are very versatile in their composition and form gels both covalently and physically. Exemplarily, covalently crosslinking has been studied by Jung et al., who provided cell‐adhesive Janus PU microfibers for tissue engineering applications. For this purpose, a commercially available, photocurable PU oligomer solution with crosslinker (NOA63) is used, that solidify via radical polymerization through UV irradiation.^[^ ^142^ ^]^ Physical crosslinking in turn has been mainly utilized in bioprinting applications. For instance, Lin et al. have synthesized a biodegradable and thermosensitive PU/soy protein hybrid bioprinting ink for direct 3D cell printing, which undergo a sol–gel transition with increasing temperature up to 37 °C.^[^ ^143^ ^]^ And Hsieh et al. synthesized a bioink based on PU nanoparticles and gelatin, which form a double network in two stages through chelation of both components at room temperature (using Ca^2+^ ions) and subsequent thermal gelation of gelatin at 37 °C.^[^ ^144^ ^]^ A combination of both, physical and covalent gelation, was provided by Hsiao et al., who synthesized a UV‐ and thermosensitive bioink based on PU nanoparticles with acrylate functionalization and thermosensitive oligodiols.^[^ ^145^ ^]^ The wide range of further gelation strategies cannot be covered completely in this review and reference is made to previous work.^[^ ^146^ ^]^

#### 4.1.2 Adaptivity: Tunability of Mechanical Material Properties

**Figure 4:**
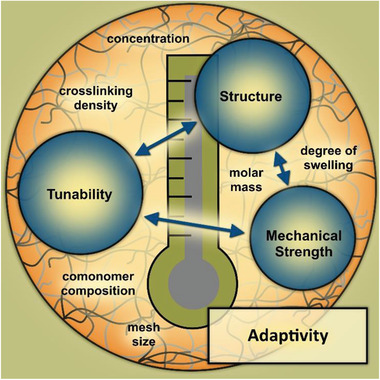
Adaptivity of materials described by the triangular relationship between nanoscopic material structure, mechanical strength, and potential tunability.

Another important requirement of materials for microtissue engineering applications is their mechanical adaptivity and tunability to the different structural conditions of in vivo tissues to ensure the best possible mimicry of host structures and thus increase the success rate with respect to animal avoidance. In vivo tissues are naturally subject to a wide range of mechanical strengths. For example, brain tissue exhibits a relatively small elastic modulus of <0.1 kPa, whereas tendons exhibit a relatively large elastic modulus of ≈1.4 MPa.^[^ ^147^ ^]^ To account for this diversity, the influence of nanoscopic material structure on the resulting macroscopic properties must be considered. Accordingly, the focus in the following is on nanoscopic material structures, their effects on mechanical strength and their potential tunability, which will be discussed in terms of mesh size *ξ*, comonomer composition, molar mass, concentration, degree of swelling and crosslink density (**Figure** **4**).

Since the material strength is often provided in the literature as shear storage modulus *G*′ [Pa] or elastic (Young's) modulus *E* [Pa], this review will focus on exactly these two mechanical parameters. Both quantities are related by the Poisson's ratio *ν* (*E* = 2*G*(1 + *ν*)). In case of incompressible materials such as hydrogels, the Poisson's ratio is usually 0.5. Hence, the shear storage and elasticity modulus can be transformed into each other according to the relationship *E* ≈ 3*G*.^[^ ^148^ ^]^

The mesh size *ξ* (distance between two network points) of a polymer network is crucial for tuning its mechanical properties. Its relationship to the mechanical material strength can be derived from the phantom network model, described by *ξ* = ((*RT*)/(*G*′ · *N*_A_))^1/3^, with *R* being the gas constant, *T* the temperature, *N*_A_ the Avogadro constant, and *G*′ the storage modulus.^[^ ^148^, ^149^, ^150^, ^151^ ^]^ Exemplarily, PEG enables easy tuning of the mesh size and mechanical strength of a corresponding hydrogel by varying the molar mass, geometric composition (linear‐, 4arm‐, 8arm‐, star‐PEG), and concentration of precursor polymers. In one example, degradable vaterite/PEG‐composite microgels were developed as in vitro models for bone tissue engineering applications. Herein, the molar mass of precursor polymers was varied to obtain shear storage moduli between 20.9 and 30.3 kPa.^[^ ^138^ ^]^ A second example was given by de Laporte and coworkers, encapsulating cells in rod‐like (anisometric) PEG‐based microgels for mimicking elongated tissue architectures such as musculoskeletal tissues. Thereby, the microgels differ in the concentration (2–5% (w/v)) of initially used precursor polymers and show storage moduli between 0.1 and 7.2 kPa.^[^ ^137^ ^]^

The mechanical strength of covalently crosslinked *PNIPAAm* hydrogels is temperature dependent, since PNIPAAm hydrogels have a volume phase transition temperature (VPTT) of 34 °C, which corresponds to the LCST of PNIPAAm polymers as described in the previous section.^[^ ^96^ ^]^ Therefore, as the temperature increases, the hydrogel displaces water for thermodynamic reasons, resulting in a decrease in hydrogel volume. This leads to a higher density of network strands per unit volume and thus to an increasing mechanical strength of PNIPAAm hydrogels with increasing temperature.^[^ ^152^ ^]^ Möhwald and co‐workers for example have developed PNIPAAm microgel films for bioapplications, which show an increase of elastic modulus from 86 to 330 kPa with increasing temperature from 25.3 to 37.2 °C.^[^ ^153^ ^]^ To further shift the VPTT of PNIPAAm and hence decrease or increase its mechanical strength at a given temperature (e.g., at physiological temperature) hydrophilic or hydrophobic comonomers can be incorporated into the PNIPAAm network.^[^ ^50^, ^95^ ^]^ Additional influences on the VPTT as pH and salt ions of the cell culture medium need to be considered as well.^[^ ^50^ ^]^

Thermoplastic and thermoset *PUs* contain hard (diisocyanate and chain extender) and soft (polyol) segments that micro‐segregate due to physical interactions between urethane groups and affect the mechanical properties of PUs.^[^ ^99^ ^]^ Correspondingly, by varying the portion of hard and soft segments in PU polymer networks, different elastic moduli could be obtained. For example, Mi et al. have synthesized soft and hard thermoplastic electrospun scaffolds for bone tissue applications and investigated the effects of incorporated hydroxyapatite (HA) particles on the mechanical strength of the polymers. In case of soft thermoplastic PUs, HA particles show no effect on the mechanical stiffness, which remains at about *E* = 0.01 MPa. By contrast, hard thermoplastic PUs exhibit a larger elastic modulus of 0.4 MPa, which, however, decreases with the incorporation of HA particles.^[^ ^154^ ^]^ Much softer PU polymers were obtained by Hill et al. who prepared PEG‐based colloidal microgel particles by self‐assembly and obtained elastic moduli between 7.5 and 2525 Pa by varying the molar mass of the soft segment PEG (2000 down to 600 g mol^−1^).^[^ ^155^ ^]^ These both examples demonstrate the wide range of possible mechanical strengths that PUs simply achieve by the composition and ratio of soft and hard segments in the polymer backbone.

By contrast, most natural materials have usually weak and less tunable mechanical properties, such as *collagen* (*G*′ = 0.5–429.7 Pa for 0.5–5.0 mg mL^−1^), *gelatin* (*E* = 179 Pa at a concentration of 10%), and *agarose* (*E* = 3.6 kPa at a concentration of 1%), which can be improved by increasing the molar mass and the concentration of precursor polymers.^[^ ^124^, ^156^, ^157^ ^]^ The mechanical strength can further be improved through the formation of interpenetrating networks with mechanical stronger polymers, as described by Ort et al. For this purpose, they have synthesized collagen/alginate‐based microgels and analyzed the influence of increasing alginate concentration (0–1.6 mg mL^−1^) on the storage modulus (0.5–2.3 kPa) of microgels.^[^ ^158^ ^]^ The mechanical properties can be further improved by chemical crosslinking. Lee et al. for example have encapsulated MCF‐7 cells and cocultures of MCF‐7 and macrophages in covalently crosslinked gelatin (GelMA)‐based microgels to provide a strategy to design tumor spheroids. They varied the precursor concentration (6–14% (w/v)) and analyzed the effect of mechanical properties (*G*′ = 1.8–18.5 kPa) on the spheroid growth.^[^ ^159^ ^]^ The stiffness of agarose can further be reduced by aldehyde‐functionalization as described by Yamada et al. for tissue engineering applications. Without aldehyde functionalization agarose‐based microgels show a storage modulus of 11.1 kPa, whereby CHO functionalization reduce the storage modulus to 0.5 kPa (both 1 wt%).^[^ ^76^ ^]^

Also *hyaluronic acid* has weak mechanical properties (*E* = 1.5 kPa at a concentration of 1%) that can be improved by chemical crosslinking.^[^ ^157^ ^]^ For example, Jooybar et al. developed hyaluronic acid‐based microgels embedded in a hydrogel for the delivery of platelet lysate (a blood product with a high concentration of growth factors). In this process, hyaluronic acid was modified with tyramine, which enzymatically crosslinks in the presence of hydrogen peroxide and horseradish peroxidase, resulting in microgels with a mechanical strength of 5.4 kPa.^[^ ^160^ ^]^ In addition, hyaluronic acid has another special feature at pH 7: At this pH, the [–COOH] units are deprotonated, resulting in a strongly hydrophilic polymer character and a water absorption up to 1000 times its solid volume.^[^ ^128^, ^131^ ^]^ In these cases, it is obvious that a high water content reduces the mechanical strength of hydrogels. A general characteristic parameter describing the water content in a hydrogel beyond the scope of hyaluronic acid is the degree of swelling. It is defined as the mass‐swelling ratio *Q*_M_ = *m*_s_/*m*_d_ between the mass of the swollen m_s_ and the dried hydrogel *m*_d_.^[^ ^161^ ^]^ Or it is defined as the volumetric swelling ratio *Q*_V_ = 1 + *ρ*_d_/*ρ*_s_ (*Q*_M_ − 1) with *ρ*_d_ and *ρ*_s_ the density of the dried and the swollen hydrogels.^[^ ^161^, ^162^ ^]^ Corresponding thermodynamic studies have been performed, for example, by Bystroňová et al. who created a 3D microenvironment based on gelatin and hyaluronic acid hydrogels suitable for in vitro microtissue modeling applications. By varying the ratio of polymer components and the crosslinking mode, different degrees of swelling and, accordingly, different mechanical strengths between 10 and 20 kPa (4% (w/v)) could be obtained.^[^ ^163^ ^]^ To relate the swelling behavior or the mechanical strength of hydrogels to the structural composition of the nanoscopic network, the Flory–Rehner equation can further be used. From this, the expression *ξ* = 0.1748(*M*_c_)^1/2^(*Q*_V_)^1/3^ can be derived, which relates the mesh size of hyaluronic acid‐based hydrogels (*ξ*) to the strand molar mass between two crosslinking points (*M*_c_) and the volumetric swelling ratio (*Q*_V_). This expression applies specifically to hyaluronic acid‐based systems, but can be adapted to other materials by considering the derivation of Collins et al.^[^ ^162^ ^]^

In turn, the mechanical strength of *alginate* (*E* = 3.6 kPa at a concentration of 1%) can further be improved by surface modification with positively charged polyelectrolytes as poly(ethylene imine), chitosan or poly‐(l‐lysine) (PLL), which adhere to the negatively charged alginate backbone.^[^ ^157^ ^]^ The change of the total surface charge restricts its swelling behavior and promotes the mechanical polymer strength.^[^ ^164^, ^165^ ^]^ Pasqua et al. for example have synthesized cell‐laden alginate‐PLL‐based microbeads for extracorporeal liver supply. Herein, PLL reinforced the mechanical stability of pure alginate microbeads, whereby the elastic module increased from ≈1 to 5 kPa with PLL modification.^[^ ^166^ ^]^

Due to its LCST slightly above the physiological temperature range (LCST = 40–50 °C), *methyl cellulose* is usually viscous at physiological conditions and has a correspondingly low mechanical strength.^[^ ^107^ ^]^ An increase in viscosity could be improved by increasing the polymer concentration and the molar mass of the polymer strands.^[^ ^86^ ^]^ Additionally, to promote conversion of the methyl cellulose solution into a gel‐like state under physiological conditions, precursor polymers with a higher proportion of methyl groups in the polymer backbone could be used. These cause an increase in hydrophobicity of the material shifting the LCST to lower temperatures.^[^ ^167^ ^]^ In combination with other polymers like alginate, methyl cellulose also forms stable hydrogels. For example, Babu et al. synthesized alginate‐methyl cellulose microspheres for drug delivery applications, whereby alginate and methyl cellulose were connected to each other by glutaraldehyde.^[^ ^168^ ^]^

#### 4.1.3 Biocompatibility: Cell Viability versus Material Functionality

Another important parameter is the biocompatibility of the materials used. These materials must actively support cell viability in the experiments through their microscopic structureand chemical functionality. Regarding the microscopic structure, access to oxygen and nutrients plays a particularly important role. Without these components, cell viability is considerably reduced, which can result in necrotic tissues and, correspondingly, the validity of the in vitro models is considerably diminished.^[^ ^169^ ^]^ Hydrogels are generally accessible to diffusible species due to their swelling behavior and porosity. However, there is often a lack of oxygen and nutrients because their diffusion is restricted in the tissue to be imitated. Usually, such diffusion occurs over a tissue thickness of 100–200 µm, beyond which it is limited.^[^ ^169^ ^]^ Due to this fact, microscopic‐scale model systems are particularly suitable for in vitro applications, as they allow oxygen and nutrient exchange by simple diffusion. In contrast, diffusion in larger constructs is limited, which can be compensated by active vascularization in the hydrogel systems. Agarwal et al. demonstrated corresponding studies by assembling microscopic tissue building blocks, together with vessel‐specific cells into macroscale vascularized 3D tumors for anticancer studies.^[^ ^170^ ^]^ Other experiments address oxygen and nutrient exchange through precise and controlled mass transport.^[^ ^171^ ^]^ For example, Ahmeda et al. cultured spheroids in bioreactors exposed to a fluid flow to enable continuous oxygen and nutrient exchange.^[^ ^172^ ^]^ A combination of both, mass transport and vascularization, is offered by Hsu et al. who have developed an organ‐on‐a‐chip assembly based on connected microtissues exposed to fresh fluid via a controlled circuit.

**Figure 5:**
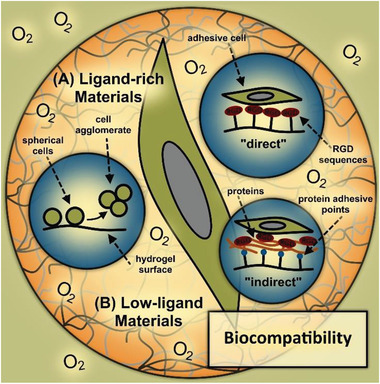
Biocompatibility of materials in microtissue engineering, with emphasis on oxygen/nutrient exchange and cell adhesion to A) ligand‐rich (“direct” vs “indirect”) and B) low‐ligand materials.

In addition to good oxygen and nutrient supply, adherent cells are essential for cell viability, proliferation, and differentiation in in vitro models. For this reason, the relationship between cell adhesion and chemical functionality of materials requires special attention. To enable cell adhesion, cells present integrins on their cell membrane that enable cells to bind to specific ligands of materials and other cells through physical and (bio‐)chemical interactions, which actively control the cell morphology, as sketched in **Figure** **5**.^[^ ^98^ ^]^ If a material is rich in ligands, cells will predominantly adhere to that material (A). These materials are accessible in two ways: via materials with the molecular structure of the ligands (“direct” cell adhesion), or via materials to which ligand‐rich materials adsorb by physical interactions (“indirect” cell adhesion). Conversely, cells exhibit a spherical morphology when a material has few cell‐binding ligands. In this case, cell–cell interactions are favored, leading to the formation of cell agglomerates and spheroids (B).^[^ ^173^, ^174^ ^]^

Proteins from the ECM with specific amino acid sequences as for example the Arg‐Gly‐Asp (RGD) sequence in fibronectin, or the Gly‐Phe‐Hyp‐Gly‐Glu‐Arg (GFOGER) sequence found in collagen, are ligand‐rich materials that enable “direct” cell adhesion.^[^ ^55^, ^175^ ^]^ Cell surface integrins link to these ligand‐rich domains by physical interactions, which is why cells adhere well to *fibronectin*, *collagen type I*, and *gelatin* based materials.^[^ ^55^ ^]^ Hence, they are widely used in in vitro models, especially in lab‐on‐the‐chip applications. According to 3R regulations, Sfriso et al. studied the interplay between endothelial cells and the plasma cascade system for cardiovascular research. Here, they used a closed microfluidic circulation system with cells cultured as monolayers in round printed polydimethylsiloxane (PDMS) microchannels that had been treated with fibronectin or type I collagen to imitate blood vessels.^[^ ^176^ ^]^ Wang et al. used a decellularized liver matrix/gelatin methacryloyl‐laden microfluidic device to mimic continuous 3D metastatic cancer cell growth as a platform for effectively testing therapeutic strategies.^[^ ^177^ ^]^

However, the structure of many materials does not allow for “directly” adhering cells due to the lack of cell‐adhesive ligands; instead, cell adhesion sometimes occurs “indirectly” through ligand‐rich components such as proteins and polypeptides that adsorb to materials with low ligand content due to their net positive or negative charge.^[^ ^98^ ^]^ Protein adsorption to these materials is usually achieved by incubation in protein‐rich cell culture medium (e.g., by adding fetal bovine serum) or by cells that actively secrete proteins.^[^ ^178^ ^]^ A corresponding ligand‐poor material to which proteins and polypeptides adsorb in a temperature‐dependent manner is *PNIPAAm* with [–CO–NH–] functionality. Above the VPTT of 34 °C, PNIPAAm materials are in the collapsed state with low water content and high protein adsorption, resulting in good cell adhesiveness. However, with decreasing temperature, the PNIPAAm material becomes more hydrated, which counteracts protein adsorption and leads to cell detachment.^[^ ^179^ ^]^ Takahashi et al. took advantage of this property to enable scaffold‐free microtissues by culturing astrocytes and neurons on PNIPAAm‐based substrates at 37 °C. After several days of cultivation, the temperature was lowered to room temperature, enabling the growth of microtissues with diameters of 50, 100, and 200 µm to detach from the PNIPAAm substrate.^[^ ^180^ ^]^ The temperature‐dependent cell attachment character of PNIPAAm hydrogels can also be rendered temperature‐independent and be stabilized by additional surface modification, e.g., with polydopamine (PDA), since PDA (a component consisting of catechol and amino groups) physically binds to both, proteins and PNIPAAm hydrogels. Accordingly, PDA‐coated PNIPAAm‐based microgel templates were fabricated as in vitro models to allow for homogeneous and temperature‐independent cell coating and adhesion on microgel surfaces to potentially mimic the blastula in embryogenesis, mammary glands, or alveolar epithelium.^[^ ^152^ ^]^ Also, some types of *PU* materials enable protein adsorption due to hydrogen bonding between urethane groups and proteins as described by Sheikholeslam et al. and Chernonosova et al.^[^ ^181^, ^182^ ^]^ Both references included PU/gelatin composite materials for tissue engineering applications prepared by electrospinning, which showed good cell adhesion on these materials.

In turn, materials based on ethylene glycol [–(CH_2_CH_2_O)*_n_*] units as *PEG* are low‐ligand materials without the potential to bind physically to ligand‐rich proteins and polypeptides, which is why they are also referred to as bioinert.^[^ ^173^, ^174^ ^]^ Accordingly, the cells do not adhere to these materials and form cell agglomerates. This property is often deliberately exploited, for example, to generate cell spheroids, as by Siltanen et al. who encapsulated hepatocytes in PEG‐based microgels by the droplet‐based microfluidic technique to generate spheroids for hepatotoxicity screening in the preclinical drug development.^[^ ^150^ ^]^ However, to further increase the cell adhesive properties of these materials, bioactivation via covalent modification with RGD or peptide sequences is possible.^[^ ^55^, ^92^, ^183^ ^]^ Corresponding RGD‐treated hollow PEG‐based micromodules were fabricated by Wang et al., where PEG diacrylate components were photopolymerized with acryloyl‐PEG‐Arg‐Gly‐Asp‐Ser sequences. These micromodules were then assembled to form a tissue‐specific morphology (e.g., liver lobules) with a vessel‐like lumen for tissue regeneration.^[^ ^133^ ^]^ Similar low‐ligand properties apply to most polysaccharides due to their hydrophilic nature.^[^ ^184^, ^185^ ^]^ In particular, *agarose* hydrogels are frequently used as a bioinert template for the synthesis of cell spheroids and organoids, as described by Gong et al., Janjić et al., and Oberoi et al.^[^ ^186^, ^187^, ^188^ ^]^ To improve cell adhesion to these materials, RGD or polypeptide units need to be incorporated into these networks as well.^[^ ^76^ ^]^ The same applies to other polysaccharide‐based hydrogels such as *alginate* and *hyaluronic acid*.^[^ ^189^, ^190^, ^191^ ^]^

#### 4.1.4 Stability/Degradability: Influences of Physiological Parameter Space

**Figure 6:**
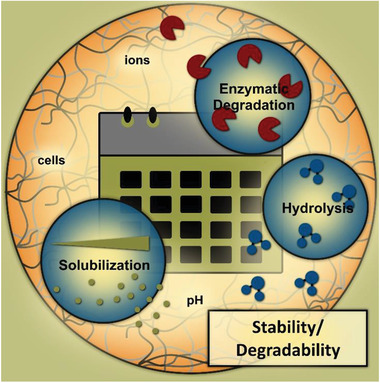
Natural stability/degradability of hydrogels to enzymes, hydrolysis, and solubilization, and key elements for control.

Long‐term stable materials are an important prerequisite for many in vitro models to achieve reproducible results without changes in the microenvironment and without the risk of toxic degradation products. Cullen and coworkers have worked exemplarily on the development of axonal pathways to modulate neuronal circulation. Here, the columnar hydrogel template must be stable until the axonal pathways have connected to host neurons via synapses.^[^ ^192^ ^]^ In contrast, there a large number of in vitro applications exist that require targeted material degradation, e.g., for the release of cells and drugs.^[^ ^193^ ^]^ Accordingly, the natural stability and degradability of materials to enzymes, hydrolysis or solubilization, as well as potential control elements for (de‐)stabilization, are presented below (**Figure** **6**).

Proteins such as *collagen* and *gelatin* are enzymatically degradable by cleavage of C—O, C—N, and C—C bonds.^[^ ^194^ ^]^ A corresponding enzymatic degradation of modified cell‐laden collagen type I microgels was analyzed by Thomas et al. by adding the enzyme collagenase. Here, the physically crosslinked microgels (≈1 mm diameter) were completely degraded within 24 hours. They also analyzed PEG‐ and glutaraldehyde‐crosslinked microgels, which degrade more slowly compared to the physical crosslinked microgels, indicating a stabilizing effect of covalent bonds.^[^ ^195^ ^]^ However, even without the addition of enzymes, protein gels could be degraded by cellular secretion enzymes, which needs to be taken into account if long‐term stable materials are desired.^[^ ^196^, ^197^ ^]^ However, because cells also produce proteins at the same time, hydrogels are often biologically remodeled, which is hugely important for cell organization, alignment, and migration in microtissues and for mimicking the natural ECM.^[^ ^198^ ^]^ Such remodeling of cell‐coated collagen microgels, for example, has been investigated by Crampton et al. as potential in vitro high‐throughput platforms. They analyzed the difference in collagen remodeling between physically (“soft”) and covalently (“stiff”) crosslinked microgels and found significant remodeling of “soft” by second harmonic generation but little to no remodeling of “stiff” microtissues.^[^ ^199^ ^]^ A corresponding dependence of materials degradation or remodeling on crosslinking density was also shown by Nichol et al. The authors synthesized GelMA‐based microgel units and analyzed the cell behavior as a function of polymer concentration (5, 10, and 15%), while cell migration and microgel remodeling decreased with increasing gel concentration.^[^ ^198^ ^]^ In summary, physical crosslinking promotes the degradation and remodeling of proteins, while covalent crosslinks often slows down these processes. The more covalent crosslinking units, the slower the degradation process and the more stable are the materials.

Enzymatic degradation is also a suitable tool for material manipulation of polysaccharides, whereby glycosidic bonds between sugar units are cleaved. Correspondingly, microtissues based on *methyl cellulose*, *agarose*, and *hyaluronic acid* are degradable in the presence of enzymes (such as cellulase, agarose, and hyaluronidase). These processes can be influenced and slowed down by their molar mass, the type of chemical modification and the degree of functionalization.^[^ ^86^, ^200^, ^201^, ^202^ ^]^ In addition to enzymatic degradation (e.g., by lyase), *alginate* (and other physically crosslinked polysaccharides and proteins) solubilizes in water due to its physical crosslinking nature, which can be controlled among others by the pH of the surrounding solvent (alginate is stable between pH 5 and 10).^[^ ^72^, ^203^ ^]^ The addition of complexing agents such as citric acid or EDTA, can further promote the liquefaction of alginate, by chelating the calcium ions of the alginate network and dissolving alginate strands.^[^ ^165^, ^204^ ^]^ Because of this property, it is often used as a sacrificial template, such as described by Nadine et al. who liquefied cell‐laden microcapsules for various tissue engineering applications using EDTA.^[^ ^165^ ^]^ By contrast, the long‐term stability of alginate can be significantly improved by covalent incorporation of crosslinkers or physical interaction with cationic polymers (e.g., chitosan).^[^ ^52^ ^]^ Yao et al. for example fabricated stable alginate‐based multicomponent fibers for cell coculture. Here, the microfibers were stabilized over a period of 21 days in cell culture medium by the addition of positively charged chitosan.^[^ ^205^ ^]^

Without the intentional incorporation of hydrolytic cleavable groups, synthetic hydrogels such as *PEG* and *PNIPAAm* are in general stable and suitable for long‐term in vitro studies of synthetic microtissues. For example, Haag and coworkers have synthesized nondegradable polyglycerol‐based microcapsules by combining the SPAAC click reaction with droplet‐based microfluidics. These platforms are used as potential cell therapeutics for long‐term isolation and protection of encapsulated cells from immune responses of potential hosts.^[^ ^206^ ^]^ In contrast, for a controlled degradation of these synthetic materials, they must be modified. For example, the chemical incorporation of peptides or polysaccharide units into these hydrogels enables enzymatic degradation. Accordingly, Rose et al. have resembled the ECM by embedding anisometric PEG‐based microgels into PEG/peptide hydrogels, which are sensitive to metalloproteinases and hence enzymatic degradation.^[^ ^207^ ^]^ Furthermore, Sattari et al. have synthesized micro‐/nanohydrogel composites based on PNIPAAm graphene oxide and starch as biodegradable crosslinker for biocompatible drug delivery.^[^ ^208^ ^]^ Another possibility to tune network degradability is the deliberate incorporation of hydrolysis‐sensitive ester groups, which can degrade into carboxylic acid and alcohol units. Accordingly, Steinhilber et al. have synthesized a hydrolysis‐sensitive PEG‐based microgel construction kit for the pH‐controlled release of living cells.^[^ ^136^ ^]^ And Sivakumaran et al. have prepared hydrolytic degradable thermoresponsive PNIPAAm‐based microgels via the microfluidic technology, using aldehyde‐ and hydrazide‐functionalized PNIPAAm precursor polymers.^[^ ^209^ ^]^ Overall, synthetic materials such as PEG and PNIPAAm in particular are easily and specifically adaptable in terms of degradability and long‐term stability through chemical modification, which makes them particularly interesting as materials for in vitro model systems from this point of view.

*PU*‐based materials, in turn, inherently contain hydrolytically labile groups in their polymer backbone, which generally makes them susceptible to degradation.^[^ ^99^ ^]^ In this context, the degradability of these materials, or conversely their stability, depends on the hydrophilicity of the hard and soft segments of the PU structure. The more hydrophilic the structure is overall, the more aqueous medium can be absorbed (increasing degree of swelling) and the faster the material degrades. Accordingly, the stability of the material can be promoted by increasing the hydrophobic content in the materials. Relevant studies were performed, for example, by Nair et al., who incubated poly(ester‐urethane)urea‐based microfiber structures in PBS (pH 7.4) at 37 °C and analyzed their degradation rate over a period of 180 days.^[^ ^210^ ^]^ Depending on the structural composition of the PU backbone, pH‐assisted degradation is also feasible, as described by Bachelder et al.^[^ ^211^ ^]^ In this work, they prepared PU microparticles for protein‐based vaccines with dimethyl acetal moieties in the polymer backbone that hydrolyze under acidic conditions.

### 4.2 Advanced Materials

To further advance the diversity and quality of in vitro model systems as a basis to replace animal experiments with high‐quality and reproducible alternatives, materials will have to become even more intelligent in the future. Building on the multiparametric material requirements discussed so far, preprogrammable material properties such as *self‐assembly*, *self‐healing*, and *4D structure* will play an increasingly important role in enabling predefined and material response functions. Often referred to as smart materials, these polymers respond in a self‐determined manner to external influences and selectively change their composition, structure, and mechanical properties. They dynamically adapt to external conditions, enabling even more concrete mimicry of in vivo tissues than general multiparametric materials.^[^ ^215^, ^216^, ^217^ ^]^ The more precisely synthetic materials adapt to natural structures and processes, the better the informative value of in vitro model systems will ultimately be and the faster animal testing can be replaced by artificial alternatives.

In this context, especially functional, synthetic, supramolecular polymers and their application in tissue regeneration are increasingly being explored.^[^ ^218^ ^]^ Modeled after natural materials, they are capable of *self‐assembly* through physical interactions, enabling the construction of complex structures with sophisticated geometric and architectural control over the entire scale. For example, Khademhosseini and coworkers have developed preprogrammable and controllable PEG‐/DNA‐based hydrogel cuboids that self‐assemble into complex microstructures through supramolecular binding interactions between complementary DNA strands.^[^ ^219^ ^]^ These microstructures form cleverly and complex assembled tissue imitations whose physicochemical and mechanical properties are comparable to those of conventional multifunctional polymer materials.^[^ ^215^ ^]^ Further insights to that topic can be found in a review article by Ouyang et al. that addresses various bottom‐up strategies for assembling building blocks in tissue engineering.^[^ ^220^ ^]^

Similar, preprogrammed properties are also exhibited by *self‐healing* materials, which can self‐heal independently and automatically to return to their normal state. This capability enables the restoration or maintenance of the original material properties and accordingly leads to an increase in the lifetime as well as the reliability of in vitro model systems.^[^ ^221^ ^]^ Mealy et al. have presented exemplary injectable, shear‐thinning, and self‐healing hyaluronic acid‐based granular hydrogels for biomedical applications, whose self‐healing properties enable rapid material stabilization after bioprinting.^[^ ^222^ ^]^ Self‐healing is mostly based on noncovalent interactions and occurs in the example through guest–host bonding between adamantane‐modified hyaluronic acid hydrogels and linear cyclodextrin‐modified hyaluronic acid strands.

The transition from static 3D materials to adaptive and responsive materials is further enabled by dynamic, preprogrammed *4D structures*, where form, function, and properties change over time due to external factors.^[^ ^223^, ^224^, ^225^ ^]^ These materials adapt to the environment during their life cycle and can be broadly described as 3D materials, taking time into account as a fourth dimension.^[^ ^226^, ^227^ ^]^ In this context, cells and materials are not considered as separate components (as it is the case in 3D structures), but as a whole, living construct that communicates with each other during cell growth and material adjustment. A relevant example is provided by Apsite et al. who used a 4D biofabrication method to produce microtissues for skeletal muscles. Herein, electrospinning is applied to develop two‐layer mats of anisotropic methacrylated alginate fibers (outer layer) and polycaprolactone nanofibers (inner layer) that self‐fold into tubes in aqueous buffer solutions. Depending on the orientation of the polycaprolactone nanofibers and the concentration of Ca^2+^ ions in the buffer solution, the tubes can be oriented differently, controlling cell growth accordingly.^[^ ^228^ ^]^ Another possibility is 4D shape memory materials (materials that return to their original shape after mechanical deformation).^[^ ^229^ ^]^ For example, cell‐loaded hollow tubular microstructures based on chemically crosslinked alginate‐methacrylate hydrogels were fabricated through bioprinting by Kirillova et al. In the presence of calcium ions, the structure of the microtubes twisted due to complex formation between alginate and buffer ions. By removing calcium ions from the hydrogel matrix by EDTA, they regained their permanent structure.^[^ ^230^ ^]^ Overall, the development of 4D material–cell composites represents another important step to further close the gap between synthetic in vitro model systems and natural in vivo tissues. Through intelligent communication between synthetic tissues and cells, they are visibly approaching the complexity of natural tissues. Together with the other preprogrammable materials, they thus ultimately drive progress in the field of in vitro model systems, and thus also in the field of animal testing avoidance.

## 5 Conclusion

The aim of the review is to discuss the increasing importance of in vitro models as a substitute for animal testing, focusing on materials properties in microtissue applications. The more defined and functional the materials are, the more likely they are to mimic similar cocultures, microtissues, and organoids in vivo. Accordingly, potential processes based on bioprinting, spheroids, microfluidics, and organ‐on‐a‐chip are addressed, as well as the origin and chemical composition of relevant materials. Another focus is on multiparametric material functionality as an important basis for the successful development of in vitro models. To this end, natural and synthetic materials are discussed in terms of their processability, adaptivity, biocompatibility and stability/degradability. In addition, advanced “smart” materials that can self‐assemble and heal through preprogramming and form 4D structures are addressed. As the abundance of materials with versatile properties has become increasingly dense in recent decades, the question now arises as to what synthetic possibilities will result from this in the future, and what significance they may have for replacing animal experiments. A correspondingly promising answer can be found in the field of synthetic biology, which has gained enormous importance in recent years. In addition to the synthesis of living biomachines for the autonomous recognition of disease states in vivo and appropriate treatment, designer cells (hybrids of living cells and artificial biological units) with adjustable properties are being targeted.^[^ ^23^, ^24^ ^]^ Their independent and agile ability to act enables personalized medicine to take a major step toward the future and hence also forms one of the many promising alternatives for the replacement of animal experiments.

## Conflict of Interest

The authors declare no conflict of interest.
